# Understanding sensitivity to BH3 mimetics: ABT-737 as a case study to foresee the complexities of personalized medicine

**DOI:** 10.1186/1750-2187-7-12

**Published:** 2012-08-16

**Authors:** Vasileios A Stamelos, Charles W Redman, Alan Richardson

**Affiliations:** 1 Institute for Science and Technology in Medicine & School of Pharmacy, Guy Hilton Research Centre, Keele University, Thornburrow Drive, Stoke-on-Trent, Keele, ST4 7QB, UK; 2 University Hospital of North Staffordshire, Newcastle Road, Stoke on Trent, Staffordshire, ST4 6QG, UK

**Keywords:** ABT-737, Navitoclax, ABT-263, BH3 mimetic, Apoptosis, Personalized medicine

## Abstract

BH3 mimetics such as ABT-737 and navitoclax bind to the BCL-2 family of proteins and induce apoptosis through the intrinsic apoptosis pathway. There is considerable variability in the sensitivity of different cells to these drugs. Understanding the molecular basis of this variability will help to determine which patients will benefit from these drugs. Furthermore, this understanding aids in the design of rational strategies to increase the sensitivity of cells which are otherwise resistant to BH3 mimetics. We discuss how the expression of BCL-2 family proteins regulates the sensitivity to ABT-737. One of these, MCL-1, has been widely described as contributing to resistance to ABT-737 which might suggest a poor response in patients with cancers that express levels of MCL-1. In some cases, resistance to ABT-737 conferred by MCL-1 is overcome by the expression of pro-apoptotic proteins that bind to apoptosis inhibitors such as MCL-1. However, the distribution of the pro-apoptotic proteins amongst the various apoptosis inhibitors also influences sensitivity to ABT-737. Furthermore, the expression of both pro- and anti-apoptotic proteins can change dynamically in response to exposure to ABT-737. Thus, there is significant complexity associated with predicting response to ABT-737. This provides a paradigm for the multiplicity of intricate factors that determine drug sensitivity which must be considered for the full implementation of personalized medicine.

## Introduction

The concept of personalized medicine envisages that therapy is tailored to take into account in each patient the pharmacodynamic and pharmacokinetic factors which may affect drug safety and efficacy. In oncology, this differs from the traditional approach in which the therapy is often based on the tissue of origin of the tumor. In personalized medicine, a treatment is chosen to address the molecular defects associated with the patient’s personal disease. In principle, it appears relatively straightforward to identify genetic (or epigenetic) abnormalities in a patient’s cancerous cells and select therapy accordingly. However, simply knowing the genes affected may be inadequate without a detailed understanding of the regulation of the signalling pathways involved. An often quoted example is provided by cetuximab, a therapeutic antibody which targets the EGF receptor. Cetuximab is effective in colon cancer driven by activation of the EGF pathway but less efficacious in patients whose tumors also are activated downstream of the EGF receptor by mutation of the gene encoding RAS. Furthermore, understanding signalling pathways in detail allows the rational design of drug combinations. We anticipate that a full implementation of personalized medicine will require profound analysis of the signalling pathways involved in the drug response. We exemplify this argument by a discussion of ABT-737, together with its cogener Navitoclax (ABT-263). ABT-737 is a “tool” compound that has been widely used, whereas ABT-263 possesses superior oral bioavailability and is currently being evaluated in clinical trials. The efficacy of these drugs depends on their ability to activate the intrinsic apoptosis pathway which we review first.

## The intrinsic apoptosis pathway

**Figure 1:**
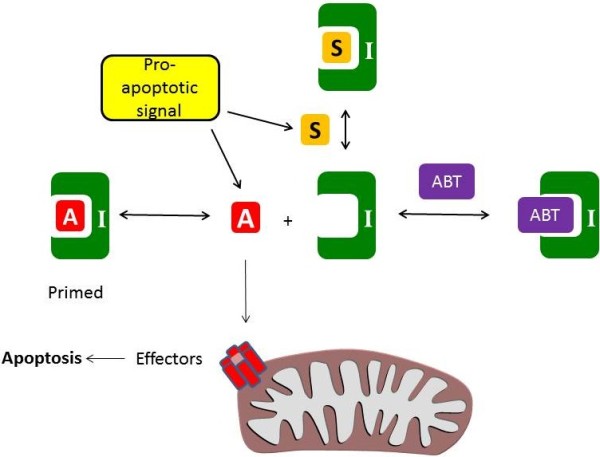
**The intrinsic apoptosis pathway. **Apoptosis is triggered by the formation of a pore in the mitochondrial outer membrane formed from the effectors. This is controlled by the activator molecules (BID, BIM, PUMA, labelled “A”) which directly stimulate the effectors (BAK and BAX). A pro-apoptotic signal (such as oncogene stress or chemotherapy) induces the expression of apoptosis activators and sensitizers. The activators may be sequestered by the inhibitors (labelled “I”, e.g. BCL-2, BCL-X_L_, MCL-1) and this prevents apoptosis. However, sensitizer molecules (labelled “S”, eg NOXA, BAD) or BH3 mimetics (eg ABT-737, labelled “ABT”) can occupy the inhibitors, preventing them from binding the activators. In cells in which the inhibitors are already primed with an activator, the effective displacement of the activator from the inhibitor by ABT-737 induces apoptosis.

The increase in oncogenic signalling (oncogene stress) and the DNA damage that occurs in genetically unstable cancer cells generate pro-apoptotic signals which, if executed, would effectively curtail tumorigenesis. Cancer cells are obliged by selective pressure to evolve mechanisms which suppress these pro-apoptotic signals. This has led to the ability of tumor cells to evade cell death through apoptosis being recognised as one of the hallmarks of cancer [1]. The dependency of cancer cells on the pathways suppressing apoptosis provides a therapeutic strategy for the treatment of cancer, because agents that inactivate these pathways should cause cell death. There are two key apoptosis pathways. The extrinsic apoptosis pathway is activated by extracellular pro-apoptotic stimuli, whereas the intrinsic pathway responds to intracellular cues. The intrinsic apoptosis pathway is controlled by the BCL-2 family of proteins (Table 1). This family comprises several classes of proteins which are considered as “effectors” (BAX, BAK), “inhibitors” (BCL-2, BCL-X_L_, BCL-2 MCL-1, BFL/A1, BCL-B), “activators” (BIM, BID, PUMA [2-4], possibly BMF and NOXA [5]) and “sensitizers” (eg BAD, BMF, NOXA). The family share several conserved “BH” domains termed BH1, BH2, BH3 and BH4. Of these domains, the activators and the sensitizers possess only the BH3 domain, and hence are often referred to as “BH3-only” proteins. These proteins are induced by cellular stress, e.g. chemotherapy. The other family members possess BH1, 2, 3 and 4 domains. The inhibitors are consequently referred to as multi-domain apoptosis inhibitors. For clarity, we shall refer to these as “apoptosis inhibitors” throughout this review, but these should not be confused with other inhibitors of apoptosis that are not part of the BCL-2 family. These multi-domain proteins possess a hydrophobic groove that serves as a binding site for BH3 domains. BAX and BAK can form homo-oligomers that form a pore in the mitochondrial outer membrane. This allows the release of several mediators of apoptosis including cytochrome C, triggering the subsequent activation of caspases. BAK normally resides in the mitochondrial outer membrane, whereas BAX translocates from the cytosol to mitochondria following activation. Two competing models have been proposed for how BAX and BAK are activated, but recent data supports a “direct activation” model in which the activator BH3-only proteins bind directly but transiently to BAX or BAK to activate them [3]. The ability of the activator BH3-only proteins to trigger BAK and BAX is restrained by the apoptosis inhibitors which sequester the BH3-only activators by binding to their BH3 domain. The apoptosis inhibitors can also suppress apoptosis by binding to the BH3 domain of the effectors and have been shown in the case of BCL-X_L_ to promote the translocation of BAX from mitochondria to the cytosol [6]. However, the apoptosis inhibitors may be prevented from suppressing apoptosis by the BH3-only sensitizers whose BH3 domain can also bind to the apoptosis inhibitors. The BH3-only sensitizers occupy the inhibitors, reducing the capacity of the apoptosis inhibitors to sequester the BH-3 only activators or the effectors (Figure 1). Thus, the BH3-only sensitizers reduce the reservoir of unliganded inhibitors available to suppress apoptosis, facilitating pro-apoptotic signalling to BAK and BAX by the BH3-only activators. The alternative “indirect” model envisages that the apoptosis inhibitors sequester the effectors. In this model, the effectors are not considered to require activation, but are continually repressed by the apoptosis inhibitors. The BH3-only proteins induce apoptosis indirectly by liberating the effectors from the apoptosis inhibitors. Variations on both these models have been proposed and it is possible that aspects of both are correct.

**Table 1:** BCL-2 family proteins

**BH3 only pro-apoptotic proteins**	**Multi-domain pro-apoptotic**	**Anti-apoptotic proteins**
*Activators*		
BIM	BAK	BCL-2
BID	BAX	BCL-X_L_
PUMA	BOK	BCL-W
		MCL-1
*Sensitizers*		BFL/BCL-2A1
NOXA		BCL-B
BAD		
BMF		
HRK		
BIK		
BNIP3		

Of the BH3 only proteins, BID, BIM and possibly PUMA are considered apoptosis activators and the others as apoptosis sensitizers. BOK is an apoptosis effector expressed primarily in the ovary.

**Figure 2:**
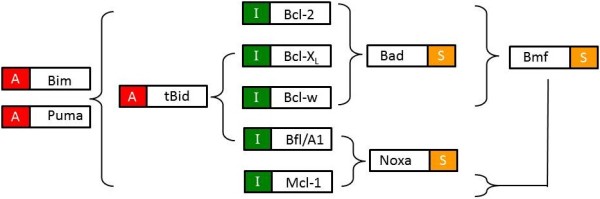
**Binding specificity of BH3 only proteins. **The activators (denoted with an “A”), BIM and PUMA bind to all the apoptosis inhibitors (denoted with an “I”). However, tBID and the sensitizers (denoted with a “S”) bind to a subset of the apoptosis inhibitors. There are some discrepancies in the literature (e.g., whether BMF binds MCL-1) and it is important to note that this diagram summarizes interactions (adapted from [7-9] in a binary fashion (interaction or no interaction) whereas in reality the interactions are better described by a range of binding affinities.

A further layer of complexity is involved because of the different binding specificity of the apoptosis inhibitors for their ligands (Figure 2). Some BH3-only proteins can bind to all the apoptosis inhibitors, whereas other BH3-only proteins exhibit specificity for a subset of the apoptosis inhibitors. For example, BAD bind preferentially to BCL-2, BCL-X_L_ and BCL-w [7,8], whereas NOXA binds preferentially to MCL-1 [7,8] and possibly BFL/A1 [7]. The BH3-only apoptosis activators BIM and PUMA are each able to bind to all the apoptosis inhibitors whereas BID has a more restricted binding profile [7,9]. Whether the induction of a BH3-only protein leads to apoptosis depends on whether the repertoire of apoptosis inhibitors expressed is adequate to dampen the pro-apoptotic signal arising from the activation of BH3-only proteins.

### ABT-737 and other BH3 mimetics

The recognition that BCL-2 family proteins regulate apoptosis has led to the development of “BH3 mimetics”, drugs which compete with BH3 domains to bind the apoptosis inhibitors. These drugs function as sensitizer molecules – although unable to directly stimulate apoptosis, they prevent the apoptosis inhibitors from sequestering the pro-apoptotic proteins through their BH3 domains (reviewed in [10]). In cells where the apoptosis inhibitors are already bound to a BH3-only apoptosis activator, the cell are considered “primed for death” because this allows a BH3 mimetic to induce apoptosis through liberation of a BH3-only activator (Figure 1). BH3 mimetics have shown remarkable single agent activity in primed cells [8] which appear addicted to apoptosis inhibitors for survival. In cells where the apoptosis inhibitors are not already primed with a BH3-only activator, exposure to chemotherapeutic agents induces BH3-only proteins that can be liberated from the inhibitors by a BH3 mimetic. Consequently, BH3 mimetics have shown synergy with a wide range of chemotherapeutic agents.

Several BH3 mimetics have been described. The best characterized is probably ABT-737 [11] and its analogue ABT-263 [12]. These compounds bind with high affinity to BCL-2, BCL-X_L_ and BCL-W but weakly to MCL-1 and BFL/A1. ABT-737 can induce apoptosis in cells in which the apoptosis inhibitors are primed with BH3-only activator proteins and ABT-737. Chemotherapeutic agents may induce priming, and consequently ABT-737 and ABT-263 are synergistic with several different chemotherapeutic agents [10,13]. Whilst the activity of ABT-737 is substantially reduced in cells lacking BAX or BAK, several BH3 mimetics other than ABT-737 retain cytotoxicity in *BAX*^−/−^/*BAK*^−/−^ cells [14,15] and unexpectedly increase the level of NOXA [16]. This suggests that the BH3 mimetics other than ABT-737 and ABT-263 possess additional activities that have yet to be fully defined. As the goal of this review is to summarize what determines sensitivity to BH3 mimetics, this undefined pharmacological activity significantly complicates a rational discussion of the factors that determine sensitivity to these drugs. Thus, this review will focus on ABT-737 (and ABT-263).

### Determinants of sensitivity to ABT-737

Knowledge of the tissue of origin of the malignant cells is not redundant, even in the era of personalized medicine, because some genetic aberrations are commonly associated with a particular cancer. For example, BCL-2 is commonly amplified in chronic lymphocytic leukemia (CLL). However, the fact that cancer is a disease involving significant genetic instability means that cancer cells sharing the same tissue origin may be genetically quite distinct. The genetic background of malignant cells can have a profound effect on drug response. Indeed, cancers of different tissue origins may share similar pharmacological sensitivities because they share common genetic anomalies. For example, chronic myelogenous leukemia and non-small cell lung cancer patients may share a polymorphism in the gene encoding BIM which leads to reduced expression of BIM isoforms possessing the BH3 domain that is necessary for its pro-apoptotic activity. Cells with this polymorphism are resistant to tyrosine kinase inhibitors, but this can be overcome with ABT-737 [17].

#### BCL-2, BCL-X_L_ and BCL-W

The most obvious determinant of sensitivity to any drug is whether cells express the intended targets. In the case of ABT-737, these are BCL-2, BCL-X_L_ and BCL-W. It is often assumed that increased expression of an oncogene indicates a tumor cell’s dependence on it. Elevated expression of these apoptosis inhibitors may be necessary to tolerate elevated levels of BH3-only proteins. However, the situation is significantly more complex, because the expression of BCL-2, BCL-X_L_ or BCL-W is a necessary but not a sufficient condition to confer drug sensitivity [18,19]. Cells in which the apoptosis inhibitors are already primed with BH3-only proteins provide an example of oncogene addiction and are particularly sensitive to ABT-737. In contrast, cells in which BCL-2, BCL-X_L_ or BCL-W are expressed but which are unoccupied by either BH3-only proteins or by BAX or BAK are not likely to be sensitive to ABT-737. Thus, measurement of expression levels of BCL-2, BCL-X_L_ and BCL-W alone may be a poor predictor of sensitivity.

An added complication has recently arisen because at least two groups have noted a discrepancy between the activity of ABT-737 expected from *in vitro* experiments and that observed in live cells. The binding profile of ABT-737 suggests that it should inhibit the binding of BH3-only proteins to BCL-2, BCL-X_L_ or BCL-W. However, in cells ABT-737 appears to more readily prevent BIM from binding to BCL-2 than to BCL-X_L_ or BCL-W [20]. This might reflect the somewhat lower affinity of BIM for BCL-2. Another report suggests that the interaction of BIM with BCL-2 or BCL-X_L_ is altered by the subcellular localization of the complex, and mitochondrial BIM complexes with BCL-2 or BCL-X_L_ are relatively insensitive to ABT-737 [21]. These observations are particularly relevant to personalized medicine, because it underscores the difficulty in making predictions of drug sensitivity using measurement of protein expression and knowledge of binding interactions measured *in vitro*.

#### MCL-1 and BFL/A1

Several lines of evidence indicate that MCL-1 confers resistance to ABT-737 because of the poor affinity of ABT-737 for MCL-1. Silencing MCL-1 by RNA interference increases the sensitivity of several cancer cell lines to ABT-737 [22-32] while ectopic expression of MCL-1 can render cells resistant to ABT-737 [24,29,33]. Similarly, prolonged exposure to ABT-737 to render cells resistant to ABT-737 leads to the up-regulation of MCL-1 [34]. Short-term exposure to ABT-737 has also been reported to elevate expression of MCL-1 [16,35,36]. However, expression of MCL-1 is not always sufficient to cause resistance to ABT-737 because occupancy of MCL-1 by pro-apoptotic proteins can effectively inactivate MCL-1 [18,37] (and see below). In the context of personalized medicine, this argues that expression of MCL-1 on its own should not necessarily preclude the use of ABT-263.

It also noteworthy that some cells appear to be more dependent on MCL-1 for survival rather than on other BCL-2 family members [38], and these cells may be sensitive to a recently described MCL-1 inhibitor “Maritoclax” [39]. By causing the degradation of MCL-1, this compound has the potential to overcome some forms of resistance to ABT-737, and has shown synergy with ABT-737 [39].

Expression of BFL1/A1 may also contribute to the sensitivity of cells to ABT-737 because, like MCL-1, BFL/A1 does not bind to ABT-737 with high affinity. Knockdown of BFL/A1 increases sensitivity to ABT-737 [27] whereas BFL/A1 over-expression reduces sensitivity [40]. Resistance to ABT-737 may also lead to up-regulation of BFL1/A1 [34].

#### NOXA

The observation that MCL-1 and BFL/A1 can confer resistance to ABT-737 suggests that BH3-only proteins that occupy these apoptosis inhibitors should increase sensitivity to ABT-737.

NOXA is particularly significant because it binds to both MCL-1 and BFL/A1, the apoptosis inhibitors implicated in resistance to ABT-737. Ectopic expression of NOXA has been shown to increase sensitivity to ABT-737 [26,32,41], whereas inhibition of NOXA expression by RNA interference decreases sensitivity to ABT-737 [40,41]. By blocking MCL-1, NOXA prevents MCL-1 from sequestering BH3-only proteins that are released from other BCL-2 family members by ABT-737. For example, ectopic expression of NOXA prevents MCL-1 sequestering BIM [41]. ABT-737 itself also increases expression of NOXA in some cells [41] but whether this is a significant and frequent phenomenon is debateable because if it were it would prevent MCL-1 from conferring resistance to ABT-737.

The expression of NOXA is increased following exposure to several chemotherapeutic agents and consequently NOXA is implicated in the synergy between these drugs and ABT-737. The importance of NOXA expression is demonstrated by several observations in which synergy between ABT-737 and chemotherapeutic agents is reduced if the expression of NOXA is repressed using RNAi. Repression of NOXA reduces synergy between ABT-737 and temozolomide [42], etoposide [27,43], vinblastine [27], cisplatin [43], CPT-11 [44] and oxaliplatin [45]. NOXA is also necessary for synergy with the proteasome inhibitors bortezomib [44] and MG132 [28].

#### BIM

Early work in the field highlighted the importance of the association of BIM with BCL-2 in determining sensitivity to ABT-737 [46]. Cancer cells from a number of lymphoid malignancies show significant (IC_50_ < 100 nM) sensitivity to ABT-737 as a single agent. In these cells, BCL-2 is already primed with BIM [8,47,48]. However, if BIM is associated with MCL-1, the cells are resistant to ABT-737 unless NOXA is also expressed and occupies MCL-1 [18]. Thus, the apoptosis inhibitor to which BIM is bound as well as which other BH3-only proteins are expressed helps determine sensitivity to ABT-737. Resistance to ABT-737 may also result from reduced expression of BIM. Small cell lung cancer (SCLC) cells grown as xenografts that have developed resistance to ABT-737 show a reduction in BIM:BCL-2 complexes compared to sensitive cells [49]. These observations underline the multi-parametric nature of sensitivity to ABT-737, and emphasize the complexity of its accurate estimation in personalized medicine.

Several chemotherapeutic agents induce the expression of BIM and probably priming of the apoptosis inhibitors with BIM. ABT-737 disrupts complexes of BIM and BCL2 (in some cells, docetaxel appears to induce priming of BCL-2 with BIM), and ABT-737 and docetaxel in combination show improved activity against the same cells grown as xenografts, compared to the activity of either agent alone [50]. Similarly, the displacement of BIM from BCL-2 by ABT-737 contributes to synergy observed between ABT-737 and TRAIL [51]. Thus, the use of ABT-263 in personalized medicine in combination with other cancer therapeutics offers yet more challenges in accurately predicting patient response because of the response to drug exposure.

#### PUMA

Recent work has suggested that PUMA functions as a direct activator of BAX and BAK [2-4]. In the presence of ABT-737 to occupy apoptosis inhibitors, the expression of PUMA may be sufficient to induce apoptosis [4]. PUMA co-operates with other BH3-only activators and ABT-737 to induce apoptosis [52] while loss of PUMA reduces the apoptotic activity of ABT-737 [2,4].

#### BID

BID is a BH3-only activator whose caspase-mediated cleavage promotes its pro-apoptotic activity. ABT-737 has been shown to trigger the cleavage of BID in leukaemia [53] and neuroblastoma cells [54]. Cleavage of BID is observed following activation of the extrinsic apoptosis pathway, so how is this enhanced by ABT-737? This could reflect cross-talk between the intrinsic and extrinsic apoptosis pathways, or we speculate it may be linked to the up-regulation of the TRAIL receptor DR5 by ABT-737 [55]. From a personalized medicine perspective, this raises the question whether it is important to consider both the extrinsic as well as the intrinsic apoptosis pathway when predicting patient response to ABT-263.

#### BAD

BAD is classified as a BH3-only sensitizer, and so its liberation by ABT-737 indirectly induces apoptosis by reducing the reservoir of apoptosis inhibitors. BAD and ABT-737 bind to the same apoptosis inhibitors suggesting that expression of BAD would be anticipated to reduce the concentration of ABT-737 required to occupy BCL-2/BCL-X_L_/BCL-W. However, there is additional complexity because by preventing the association of BAD with BCL-X_L_, ABT-737 slows the turnover of BAD, leading to a striking increase in BAD protein [56]. The relative contributions of BAD prior to, and after, exposure to ABT-737 remain unclear.

#### Summary of factors affecting sensitivity

From the foregoing discussion, it is clear that there is no single determinant of sensitivity to ABT-737. In several cases, various BH3-only proteins have been identified as being “necessary” for ABT-737 to exhibit pro-apoptotic activity. Discrepancies between different studies probably reflect differences in the experimental setting: whether cells are primed; the genetic background of the cells being studied; the BH3-only proteins and the apoptosis inhibitors expressed and the effect of ABT-737 on their expression. The over-arching factor that determines whether ABT-737 induces apoptosis appears to be whether sufficient BH3-only proteins can be released to exceed the buffering capacity of the apoptotic inhibitors when cells are exposed to ABT-737. This is determined by the repertoire of apoptosis inhibitors, BH3-only activators and sensitizers expressed in a particular cell. As we will discuss below, this creates a significant dilemma for predicting drug sensitivity in the clinic. Analysis of protein expression alone may not be sufficient to understand the drug sensitivity of an individual tumor, rather measurement of the functional status of the intrinsic apoptosis pathway may be a superior predictor.

## Strategies for overcoming resistance to ABT-737

One aspect of personalized medicine envisages that patients may not receive a drug where assessment of the molecular abnormalities in their tumor predicts that the drug is unlikely to be effective. But through an understanding of the pathways regulated by ABT-737 and ABT-263, can we rationally design drug combinations which increase the sensitivity of cells to these drugs? This may benefit patients predicted to be unresponsive to ABT-263. Two general strategies have been advanced, categorized as either decreasing the expression of MCL-1, or increasing the expression of BH3-only proteins, or in some cases both of these factors are involved.

### Decreasing the expression of MCL-1

**Figure 3:**
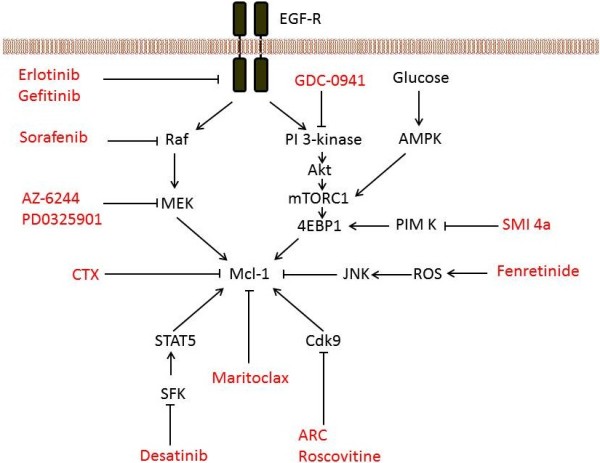
**Strategies to decrease the expression of MCL-1. **The sensitivity of cells to ABT-737 can be increased using drugs which decrease the expression of MCL-1. The ERK (including RAF and MEK) and PI 3-kinase pathways are key regulators of MCL-1 expression so drugs inhibiting this pathway show synergy with ABT-737. Drugs inhibiting other regulators of MCL-1 are also shown, and these drugs also increase sensitivity to ABT-737. ARC, 4-amino-6-hydrazino-7-beta-D-ribofuranosyl-7 H-pyrrolo(2,3-d)-pyrimidine-5-carboxamide; CTX, chemotherapy; EGF-R, EGF receptor; ROS, reactive oxygen species; SFK, SRC family kinase. For references, the reader is referred to the main text.

The observation that MCL-1 can confer resistance to ABT-737 suggests that strategies to decrease the expression of MCL-1 might increase sensitivity to ABT-737. Several drugs have been proven effective in preclinical studies (Figure 3). This approach is also particularly important because exposure to ABT-737 can itself increase expression of MCL-1 [16,35,36] thus providing a mechanism by which resistance to ABT-737 can be caused by the drug itself.

Activation of the MAP-kinase (ERK) signalling cascade increases levels of MCL-1 through increased transcription [57] and increased protein stability [58]. Consequently inhibition of components of this cascade increase sensitivity to ABT-737. This appears to be a particularly important locus for intervention, because exposure to ABT-737 activates the MAP-kinase pathway [59], and increases expression of MCL-1 [31,34,53,60,61]. Thus, repression of the ERK pathway through inhibition of RAF with sorafenib or inhibition of MEK reduces levels of MCL-1 and is synergistic with ABT-737 *in vitro *[59,62,63]*.* ABT-737 combined with a MEK or RAF inhibitor is more effective in xenograft studies than the single agents [31,59,63].

Dasatinib is an inhibitor of BCRABL and SRC-family kinases. In haematological malignancies, dasatinib has been shown to inhibit SRC-family kinase-mediated activation of the transcription factor STAT5, and correspondingly decrease the expression of MCL-1 [40,64-66]. Dasatinib also reduces the expression of MCL-1 by inhibiting the SRC-family kinase LYN which suppresses the expression of miR-181. This microRNA recognizes the MCL-1 3’ UTR [67], decreasing expression of MCL-1 and contributes to the synergy between dasatinib and ABT-737 in CLL cells [40]. As LYN is widely expressed, it will be of interest to evaluate this strategy in other cancers. Synergy between ABT-737 and two other BCRABL inhibitors, imatinib and nilotinib, that are used in the treatment of CML has also been reported [68-71]. In part, this synergy may also reflect reduction of MCL-1 by imatinib [72]. This is of particular interest because resistance to imatinib can lead to treatment failure. Potential mechanisms of resistance to imatinib include increased expression of the apoptosis inhibitors [68,71] or diminished expression of BH3-only proteins [71]. This offers the prospect of combining ABT-263 with BCRABL inhibitors to treat CML.

The PI3-KINASE pathway is frequently activated in cancer, leading to activation of AKT and several downstream effectors. Amongst these is mTORC1 which regulates 5’ cap-dependent mRNA translation through phosphorylation of 4EBP1. This is particularly important because the short half-life of MCL-1 suggests that interfering with MCL-1 protein synthesis should have a dramatic effect on expression levels. Examples of inhibition at several points on the PI 3-KINASE/AKT/mTORC1 pathway and its impact on MCL-1 are discussed below, although it is important to note that factors other than regulation of MCL-1 synthesis may contribute to the impact on protein level.

GDC-0941 is a PI 3-KINASE inhibitor which reduces the expression of MCL-1 [35]. In part, this reflects the induction of BH3-only proteins which may promote turnover of MCL-1. GDC-941 and ABT-737 synergistically induce cell death *in vitro* and in combination inhibit xenograft growth [35]. Importantly, ectopic expression of MCL-1 reduces this effect of the drug combination. Inhibition of other members of the PI 3-KINASE signalling pathway also provides potential routes for increasing sensitivity because inhibition of AKT or mTORC1 is also synergistic with ABT-737 [73,74].

MCL-1 expression is maintained in the presence of extracellular glucose, but it rapidly diminished in its absence. Inhibition of glycolysis with 2-deoxyglucose (2-DG) can also inhibit the expression of MCL-1 [75,76]. This may in part be mediated through regulation of protein synthesis by the PI 3-KINASE/AKT/mTORC1 pathway. Inhibition of glucose metabolism and consequently mTORC1 reduces phosphorylation of 4EBP1 and decreases expression of MCL-1 [75,76]. However, activation of this pathway by constitutively active AKT is not sufficient to maintain the expression of MCL-1 in the absence of extracellular glucose [75], suggesting that glucose regulates MCL-1 expression through more than one pathway. Nonetheless, resistance to ABT-737 could be overcome by inhibition of glycolysis with 2-DG or mTORC1 with PP242 [75]. In other cells, 2-DG does not substantially reduce expression of MCL-1, but it does block its ability to form a complex with BAK [37]. Furthermore, the combination of 2-DG with ABT-737 or ABT-263 improved the survival of animals with an experimental model of lymphoma [77] or prostate cancer [37] beyond that seen in animals treated with either agent alone.

PIM kinases are also involved in the regulation of transcription, translation, cell survival and energy metabolism [78], including regulation of phosphorylation of 4EBP1. Of particular interest, inhibition of PIM kinases decreases translation of MCL-1 mRNA, as well as increasing degradation of MCL-1 protein [33]. The reduced level of MCL-1 (as well as up-regulation of NOXA, see below) is thought to underlie the more than additive activity seen with a PIM inhibitor and ABT-737 both *in vitro* and in xenograft studies [33].

CDK9 is part of the transcription elongation factor b complex that regulates elongation of mRNA [79]. ARC is a nucleoside analogue which inhibits CDK9 and reduces the expression of MCL-1 [80]. In combination with ABT-737, ARC induces apoptosis and causes a synergistic inhibition of cell survival. The transcription of MCL-1 can also be reduced by exposure to roscovitine which inhibits several cyclin dependent kinases including CDK9 and this also increases sensitivity to ABT737 [15,24,40]. Synergy with flavopiridol, another pan-CDK inhibitor that down-regulates MCL-1, has also been observed [30].

Fenretinide is a synthetic retinoid whose cytotoxic activity depends in part on the generation of reactive oxygen species (ROS). Subsequent activation of JNK by ROS leads to phosphorylation and decreased levels of MCL-1 [53]. Synergy has been observed between fenretinide and ABT-737 in acute lymphoid leukemia (ALL) and neuroblastoma cells *in vitro *[53,54] and the drug combination improves the survival of mice bearing neuroblastoma xenografts beyond that seen with either agent alone [54].

Several chemotherapeutic agents have shown synergy with ABT-737 (reviewed in [10]) and in some cases this reflects the decreased MCL-1 caused by the chemotherapy, for example by etoposide or cisplatin [43,81]. In other cases, the induction of the pro-apoptotic protein NOXA may lead to the ubiquitination and proteasomal degradation of MCL-1 [82]. However, down-regulation of MCL-1 has also been observed without an accompanying increase in NOXA [43][83], suggesting that the chemotherapeutic agents decreased the expression of MCL-1 through several mechanisms. The transcriptional inhibitor actinomycin has been shown to reduce MCL-1 [29,83]. The activity of this chemotherapeutic agent is reminiscent of the effect seen when targeted agents are used to inhibit transcription and translation through inhibition of cyclin-dependant kinases, mTORC or PIM kinases.

Other agents which have shown synergy with ABT-737 by virtue of down-regulating MCL-1 include methylseleninic acid [84] and L-asparaginase [85].

### Induction of BH3 proteins

An alternative to decreasing the expression of MCL-1 is to induce BH3-only proteins to occupy MCL-1 and hence increase sensitivity to ABT-737. Both chemotherapeutic and molecularly-targeted agents have been used successfully.

Induction of NOXA appears to be one key strategy to increase sensitivity to ABT-737, because NOXA binds to the BCL-2 family proteins MCL-1 and BFL/A1 that are insensitive to antagonism by ABT-737. Consequently, NOXA can co-operate with ABT-737 to occupy the full repertoire of anti-apoptotic BCL-2 family inhibitors. NOXA has also been reported to induce the degradation of MCL-1 [82,86,87] but not in all cells [41]. NOXA is induced by exposure to several chemotherapeutic agents including fludarabine [40], CPT11 [88], imiquimod [25], dacarbazine [25] and actinomycin [83] and in each of these cases, synergy with ABT-737 has been observed. Although temozolomide as a single agent does not appreciably affect NOXA, its combination with ABT-737 induces NOXA, leading to a more than additive induction of apoptosis and improved activity in a xenograft model [42]. In several cases, the induction of NOXA appears crucial, as inhibition of the expression of NOXA using RNA interference reduces the effect of the drug combination [25,42,44]. Similarly, ectopic expression of NOXA enhances the effect of the combination of actinomycin and ABT-737 [83].

Expression of NOXA is also increased by exposure to targeted agents including several proposed BH3 mimetics [16], bortezomib [30,40], a PIM kinase inhibitor [33], and inhibition of signalling through the NOTCH pathway using a γ-SECRETASE inhibitor [89]. NOXA appears to contribute to the synergy observed between ABT-737 and these agents because knockdown of NOXA reduces the effect of the drug combination [40,89].

BIM binds to all the known apoptosis inhibitors, including MCL-1. Inhibition of the epidermal growth factor receptor (EGFR) with erlotinib or gefitinib leads to increased expression of BIM [46,90,91], probably through transcriptional and translational regulation [90], and apoptosis. In these experiments, ABT-737 exhibited minimal activity when tested on its own, but when combined with erlotinib or gefitinib, a more than additive increase in cell death was observed. Combining ABT-737 with an EGFR inhibitor may avoid early resistance to EGFR inhibitors that arises from increased BCL-2/ BCL-X_L_ expression [92].

These data suggest that EGFR suppresses the expression of BIM. One of the downstream pathways mediating this is likely to be the ERK (MAP) kinase pathway because inhibition of RAF or MEK increased the expression of BIM. Sorafenib (which inhibits several kinases including RAF) induces BIM and apoptosis and is synergistic with ABT-737 in AML cells [93]. In AML cells that are relatively insensitive to ABT-737 alone, a MEK inhibitor was synergistic with ABT-737 [59]. This synergy was dependent on BIM because suppression of BIM by RNAi reduced apoptosis that was induced by the drug combination. A MEK inhibitor also improved the efficacy of ABT-737 in an AML xenograft model [59]. In NSCLC cells, a MEK inhibitor caused predominantly G_1_ arrest and ABT-737 on its own minimally induced apoptosis [63]. However, when the drugs were combined, a more than additive induction of apoptosis was observed and the drug combination showed substantially improved activity in a xenograft models.

An inhibitor of JAK2 induced BIM and also showed synergy with ABT-737 in cells harbouring a JAK2 activating mutation [94].

Histone deacetylase (HDAC) inhibitors have also been shown to induce expression of BIM and the preferential priming by BIM of BCL-2 and BCL-X_L_ than of MCL-1. Consequently, the HDAC inhibitor suberoyl bis-hydroxamic acid was found to induce apoptosis synergistically with ABT-737 [95]. ABT-737 released BIM from complexes with BCL-2 or BCL-X_L_, and knockdown of BIM substantially blunted the effect of the drug combinations. Vorinostat (also known as SAHA, another HDAC inhibitor) induces histone acetylation, the expression of the genes encoding BMF, BIM and NOXA and consequent priming of BCL-2 [96]. As a result, vorinostat showed synergy with ABT-737 which was reduced by inhibition of BMF expression by shRNA.

Although BAD does not bind to MCL-1, it is possible that it could still improve sensitivity to ABT-737 by reducing the total capacity of free apoptosis inhibitors. Both sorafenib [61,93] and ABT-737 [56] can increase BAD protein levels. Sorafenib increased the transcription of BAD [61] whereas ABT-737 decreased the turnover of BAD [56]. Sorafenib also regulates BAD through inhibition of BAD phosphorylation, promoting its pro-apoptotic activity. Sorafenib and ABT-737 have been shown to display synergistic activity in a number of cell types [61,93]. However, it is difficult to ascribe this solely to the effect of sorafenib on BAD, because of the impact of sorafenib on MCL-1.

BAD is also likely to play a role in the change in sensitivity to ABT-737 when cells are deprived of glucose or exposed to 2-DG (discussed above). In its unphosphorylated state, BAD promotes apoptosis, but when phosphorylated it regulates glucose metabolism [97]. Dephosphorylation of BAD is promoted by glucose deprivation [98], suggesting that 2-DG may do the same, and that this may contribute to the synergy observed between ABT-737 and 2-DG. However, 2-DG also induces the expression of BIM, BMF and NOXA [99,100] and glucose deprivation induces PUMA, BID and BIM [75,101], again making it difficult to assess the contribution of BAD.

## Factors that complicate measurement of sensitivity to BH3 mimetics

The underlying goal of the work discussed in the previous section was to develop methods that can be translated to the clinic to improve patients’ response to ABT-737/ABT-263. This presupposes that experimental measurements of the sensitivity to ABT-737 made in the laboratory are predictive of clinical outcome. How good are our preclinical models? While xenograft studies are considered by many to remain the best predictors of clinical activity, they are costly and relatively slow. This makes it crucially important that cellular assays are as realistic as possible so that appropriate hypotheses are tested in animal and clinical studies. Perhaps the most common setting in which sensitivity to drugs is measured uses cancer cell lines growing in nutrient and growth-factor rich media as a monolayer attached to cell culture plasticware in 21 % O_2_ (i.e. atmospheric oxygen). The past decade has seen a growing appreciation that this is perhaps not the most realistic model, although it is experimentally convenient. In patients, tumor cells may be located in hypoxic, nutrient-poor environments comprising 3-dimensional tumors. Each of these factors has been reported to affect cellular sensitivity to ABT-737.

### Metabolism

Tumor cells are thought to depend more on glycolysis than oxidative phosphorylation to provide ATP and as we have noted, the extracellular glucose concentration affects sensitivity to ABT-737. Metabolic factors that influence sensitivity are not limited to carbohydrate metabolism. Starvation of amino acids and growth factors leads to the up-regulation of PUMA [102], and its subsequent priming of BCL-X_L_ renders cells sensitive to ABT-737.

ABT-737 is further linked to metabolism through its ability to stimulate autophagy. The nucleation phase of autophagy involves the activation of the lipid kinase VPS34 by the BH3-only protein BECLIN. BCL-2 can sequester BECLIN and the release of BECLIN by ABT-737 triggers autophagy [103,104]. However, ABT-737 also activates other regulators of autophagy including AMPK and IKK, both of which appear to be necessary for ABT-737 to stimulate autophagy [104]. How these factors conspire with the pro-apoptotic activity of ABT-737 to determine the final cellular response to ABT-737 is unclear, as autophagy can promote both cell survival and cell death. Nevertheless it is clear that metabolism and the activity of ABT-737 are intimately linked.

### Hypoxia

Several reports indicate that hypoxia increases the sensitivity of tumor cells to ABT-737 [105-107]. This may reflect down-regulation of MCL-1 [105], or up-regulation of BH3-only proteins including PUMA, NOXA, BNIP3 or NOXA [107]. It is noteworthy that cells resistant to anoxia show increased expression of apoptosis inhibitors [107]. In xenograft studies, ABT-737 induces apoptosis preferentially in hypoxic regions of the tumor [105]. Finally, the extent of synergy seen between chemotherapeutic agents and ABT-737 is also dependent on oxygen concentration [105]. Thus, it may be advisable to evaluate ABT-737 activity in laboratory experiments under a more physiological oxygen tension.

### 3-dimensional culture

An alternative to testing the sensitivity of cells in a monolayer is to use 3-dimensional cultures such as spheroids. These are aggregates of growing tumor cells prevented from adhering to a solid surface and which mimic a small tumor. The repertoire of BCL-2 family proteins appears to change in cells grown as spheroids, compared to the same cell line grown as a monolayer. Increases in the expression of BCL-2 and BCL-X_L_ have been observed in spheroids [108-111], and decreases in the expression of MCL-1 has also been reported [108,109]. This implies a switch towards an ABT-737-sensitive phenotype in cells grown as spheroids. Thus, even though increased resistance to either cisplatin or the combination of bortezomib and TRAIL was observed when cells were grown as spheroids, this could be overcome by exposure to ABT-737 [108,109]. The expression of pro-apoptotic family members may also be changed, and the growth of melanoma cells in spheroids leads to reduced expression of NOXA [26] but increased expression of BIM confers sensitivity to ABT-737 [112].

Although these observations highlight the usefulness of spheroids as tumor models, some caution is appropriate A decrease in drug sensitivity in spheroid culture may also be due to poor diffusion of the drug into the spheroid inner mass [26], or a reduced rate of proliferation. The changes in the expression of both pro- and anti-apoptotic BCL-2 family members observed in spheroids may also be linked to the oxygen gradient between the inside and outside of the spheroid [105].

These observations suggest that a more complete understanding of tumor cell sensitivity to ABT-737 requires that we adapt our experimental condition. It is interesting to note that in some cases ABT-737 inhibits the growth of xenografts in animals even though this is not predicted by experiments performed under standard cell culture conditions [81,113]. We suggest that although it is reasonable to evaluate the gross factors that determine sensitivity to ABT-737 using standard cell culture conditions, a refined understanding will be provided by using more complex models that recapitulate (patho)physiological conditions more closely. The clinical evaluation of the conclusions from laboratory experiments is then more likely to have a successful outcome.

## Translational impact

Which patient groups might benefit from ABT-263? In addition to activity as monotherapy in animal models of lung cancer [12,49,114][11] and several lymphoid malignancies [11,12,15,62,85,115], ABT-737 or ABT-263 have also shown activity in combination with other drugs in animal models of numerous different cancer types. This includes the most prevalent cancers, breast [35,50], prostate [37], lung [92,116-118] and colorectal cancers [45] cancers. In principle, BH3 mimetics may improve the response to any chemotherapeutic agent that induces apoptosis through the intrinsic pathway. Thus, the use of such drugs may not be limited to cancers originating from particular tissues. Consequently, a key goal of clinical studies with ABT-263 will be to identify which patients should receive the drug, either alone or in combination with another drug. Bearing in mind the number of factors discussed above that influence sensitivity to ABT-737, how can this be achieved? There remain a number of complications which must be addressed.

Firstly, several groups have failed to demonstrate a correlation between drug sensitivity and expression of an individual apoptosis inhibitor [40,41,119,120]. The discovery that MCL-1 confers resistance to ABT-737 (and presumably ABT-263) suggests that measurement of MCL-1 could be used to exclude patients from treatment with ABT-263 and instead direct the use of ABT-263 in combination with a drug to repress MCL-1. The predictive value of MCL-1 measurement has been improved by measuring ratios of multiple ABT-737- sensitive and insensitive apoptosis inhibitors [30,120,121]. This suggests that the expression of all the BCL-2 family apoptosis inhibitors should be considered.

Secondly, the BH3-only proteins also influence sensitivity to ABT-737. Thus, correlations between NOXA/MCL-1 [40,41] and BIM/MCL-1 [73] ratios have been reported. This suggests that it would be helpful to include measurement of these in any biomarker panel.

Thirdly, even measuring the expression of both apoptosis inhibitors and BH3-only proteins may not be adequate, because some tumor cells exhibit dependency on more than one apoptosis inhibitor for survival. For example, cells containing BIM in complex with MCL-1 and BCL-2 may still be sensitive to ABT-737 if sufficient BIM can be liberated from BCL-2 to induce apoptosis [18]. Priming of the apoptosis inhibitors provides a good correlation with clinical response to chemotherapy [122] and the response to ABT-737 in primary cultures [47]. This suggests that determining the distribution of pro-apoptotic proteins among the various apoptosis inhibitors is likely to be a better predictor of sensitivity to ABT-737 than simply measuring expression [18,123]. Moreover, there are discrepancies between the sensitivities of these complexes in cells to ABT-737 and those anticipated from *in vitro* studies [20,21]. This suggests that extrapolating from knowledge of *in vitro* binding interactions of BH3-only proteins and apoptosis inhibitors to what happens in the clinical setting may be challenging. An alternative is to use a functional measure of the activity of ABT-263 in which the pharmacodynamic activity of the drug is directly measured, for example “BH3 profiling”. This provides a functional measurement of the sensitivity of cells to ABT-737 through assessment of the drug’s effect (or that of a peptide corresponding to a BH3 domain) on mitochondria isolated from cancer cells; this can also identify the apoptosis inhibitors that cells are dependent on for survival. Importantly, this assay has the potential to be used clinically [124], overcoming some of the difficulties discussed above. By directly measuring the effect of the drug, it is neither necessary to have knowledge of the expression of BCL-2 family members, nor to analyse their interactions.

Fourthly, short-term exposure to ABT-737 has also been reported to elevate expression of MCL-1 [16,35,36], BFL/A1 [34] as well as BAD [56,61] and NOXA [41]. Thus, measurement of BCL-2 family members prior to drug treatment may provide an inaccurate indicator of drug response. It may be preferable to briefly expose patients to ABT-263 prior to collecting malignant tissue (e.g. by biopsy or surgery). This will allow for any potential effects of the drug on the expression of BCL-2 family members to be assessed, prior to predicting the sensitivity of the tumor tissue to ABT-263.

Finally, we have highlighted how experimental conditions can affect the sensitivity to ABT-737. Clearly any proposed biomarkers to predict patient response requires validation in patients, but in the present case it appears to be particularly important to consider the strength of the preclinical evidence prior to advancing potential biomarkers to clinical evaluation.

These considerations raise the issue whether accurately selecting subsets of patients to receive BH3 mimetic therapy is feasible. What does the clinical experience with ABT-263 so far tell us? Preliminary evidence for efficacy has been observed in several lymphoid malignancies [125]. In a recent phase 1 trial in CLL, 34 % of patients showed a partial response to ABT-263 [126]. However, there was no correlation between BCL-2 expression and patient response, although a high BIM:MCL-1 ratio (implying occupancy of MCL-1 overcoming resistance to ABT-263) was associated with a response. Somewhat less encouraging results were obtained in patients with relapsed SCLC [127] where only 2.6 % of patients showed a partial response (although drug-induced apoptosis was more pronounced in patients thought to express BCL-2 more highly). Importantly, in both CLL and SCLC, BCL-2 expression is elevated in the majority of patients yet the outcomes of the trials are clearly distinct. Thus, these clinical studies reinforce the notion that measuring the expression of the targets of ABT-263 is likely to be an inadequate predictor of response.

Considering the difficulties in predicting response that we have discussed above, it might even be argued that there is merit in considering using ABT-263 without attempting to identify which patients groups are most likely to respond. However, the lifespan of platelets is regulated by BCL-X_L_ which consequently is shortened by ABT-737 [128,129]. Correspondingly, thrombocytopenia is a major adverse effect of ABT-263 [125-127,130] which may preclude its “non-selective” use. Patients with already reduced platelets (e.g. some leukemias) may be particularly at risk if treated with ABT-263. This also suggests the possibility that drug interactions may occur in patients co-treated with other drugs such as carboplatin that also cause thrombocytopenia. This may limit the use of ABT-263 in combination with carboplatin, which is unfortunate because synergy between ABT-737 and carboplatin has been observed in preclinical studies [113]. It is interesting to note that Abbot have recently developed ABT-199, an analog of ABT-263 that avoids antagonising BCL-X_L_ that may overcome some of these difficulties. However, this drug is unlikely to be effective in tumors dependent on BCL-X_L_ rather than BCL-2 (eg ovarian cancer, [113]) and we speculate that BCL-X_L_ will emerge as a mechanism of resistance to ABT-199.

Taking into account the complexities of measuring drug sensitivity that we have discussed, our opinion is that it would be preferable not to select patients for entry into clinical trials of BH3 mimetics using measurement of the expression of BCL-2 family members alone (even if this includes both pro- and anti-apoptotic members). Although there have been notable successes in using biomarkers to identify patients who respond to a particular drug, we doubt whether measuring expression levels alone will identify all patients likely to respond to ABT-263. However, some method of patient selection is clearly desirable. We consider that assays that measure functional outcome as the response to a drug are preferable because they are independent of our assumptions regarding factors that control drug sensitivity. One such assay is BH3 profiling. Alternatively, we note that apoptosis can be measured in patients within 6 hours of SCLC patients receiving ABT-263 [130]. We believe that this is likely to provide a more robust prediction of drug therapeutic activity because it measures the drug’s anticipated pharmacological effect in patients. Such tests evaluating the pharmacodynamic activity of ABT-263 could be used as the basis of selecting patients to receive ABT-263. In principle, this approach could be applied more widely in personalized medicine in oncology, where drug-induced apoptosis is frequently a desired outcome.

## Conclusion

The trend from cytotoxic chemotherapy towards targeted therapeutics offers the promise of improved efficacy accompanied by a reduction in the adverse effects seen with chemotherapy. Currently, cancer therapies take patient characteristics into consideration but with regards to the tumor itself, other than conventional factors such as stage and histological type, traditional therapy has been based on a ‘one size fits all’ approach. While this may have been acceptable with chemotherapy, targeted therapeutics are designed to be more selective so the possibility that tumors can evade the effects of the drug is substantially increased. The more we learn, the more we appreciate the complex heterogeneity that exists between and within tumor types. Thus, patients are likely to be more varied in their response to targeted therapies than to chemotherapy. The concept of personalised medicine is an appealing solution to this problem, but demands a depth of understanding of disease biology that is only just beginning to be translated from the laboratory to the clinic.

## Competing interests

The authors declare that they have no competing interests.

## Authors’ contributions

All the authors contribute to reviewing the literature in this review, helped to draft the manuscript and read and approved the final manuscript.

## Authors’ information

VS is a final year Ph.D student and a trainee gynaecologist with a particular interest in gynaecological oncology.

CWR is a consultant gynecological oncology surgeon with a particular interest in ovarian cancer.

AR is a senior lecturer in pharmacology who has conducted drug discovery research in the pharmaceutical industry as well as translational research in academic institutions. He has a particular interest in ovarian cancer.
